# Molecular basis for selective uptake and elimination of organic anions in the kidney by OAT1

**DOI:** 10.1038/s41594-023-01039-y

**Published:** 2023-07-23

**Authors:** Joanne L. Parker, Takafumi Kato, Gabriel Kuteyi, Oleg Sitsel, Simon Newstead

**Affiliations:** 1 Department of Biochemistry, University of Oxford, Oxford, UK; 2 The Kavli Institute for Nanoscience Discovery, University of Oxford, Oxford, UK; 3 Present Address: Max Planck Institute of Biochemistry, Max Planck Institute of Molecular Physiology, Dortmund, Germany

**Keywords:** Structural biology, Metabolism

## Abstract

In mammals, the kidney plays an essential role in maintaining blood homeostasis through the selective uptake, retention or elimination of toxins, drugs and metabolites. Organic anion transporters (OATs) are responsible for the recognition of metabolites and toxins in the nephron and their eventual urinary excretion. Inhibition of OATs is used therapeutically to improve drug efficacy and reduce nephrotoxicity. The founding member of the renal organic anion transporter family, OAT1 (also known as SLC22A6), uses the export of α-ketoglutarate (α-KG), a key intermediate in the Krebs cycle, to drive selective transport and is allosterically regulated by intracellular chloride. However, the mechanisms linking metabolite cycling, drug transport and intracellular chloride remain obscure. Here, we present cryogenic-electron microscopy structures of OAT1 bound to α-KG, the antiviral tenofovir and clinical inhibitor probenecid, used in the treatment of Gout. Complementary in vivo cellular assays explain the molecular basis for α-KG driven drug elimination and the allosteric regulation of organic anion transport in the kidney by chloride.

## Abstract (web summary)

Organic anion transporter 1 (OAT1) structures reveal the molecular basis for the selective uptake and elimination of metabolites, drugs and toxins in the kidney.

## Main

Organic anions comprise a large group of endogenous and exogenous compounds, including tricarboxylic acid intermediates, bile acids, prostaglandins, fatty acids, anionic drugs and environmental toxins. Many organic anions result from the breakdown of metabolites, such as nucleic and amino acids, and must be cleared from the body to avoid accumulation and toxicity^1,2^. The transport of organic anions across the cell membrane is mediated by the organic anion transporters (OATs), the organic anion transporting polypeptides (OATPs) and the multidrug resistance-associated family of ATP-driven transporters^3–5^. The OATs belong to the SLC22 family of solute carriers and structurally belong to the major facilitator superfamily (MFS) of secondary active transporters^3,6^. The SLC22 family consists of organic anion and cation transporters and are widely expressed in tissues involved in metabolite exchange, such as the intestine, kidney, liver and blood–brain barrier^7^. Within the SLC22 family, substrate specificity varies, with members such as OAT1 and OAT3 recognizing a wide range of anionic ligands; others are more specialized such as URAT1 (also known as SLC22A12), which is selective for uric acid^8^.

**Fig. 1:**
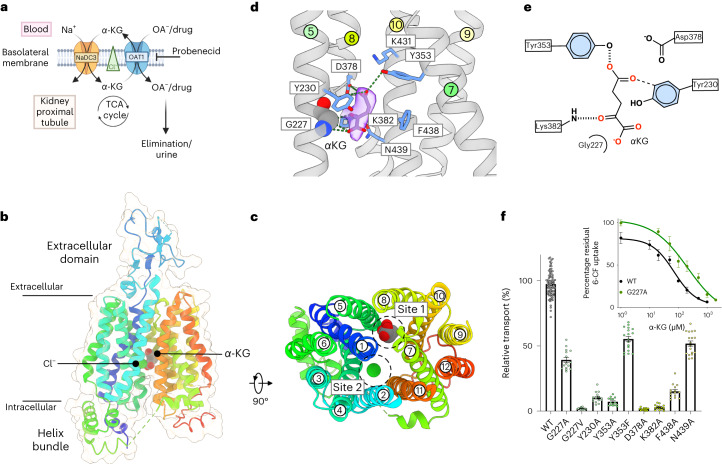
Cryo-EM structure of Oat1 bound to α-KG. **a**, OAT1 exchanges α-KG, produced from the tricarboxylic acid (TCA) cycle or transported into the cell via NaDC3, for organic anions and drugs. The transport function of OAT1 is inhibited by the drug probenecid and enhanced by chloride binding. **b**, Cryo-EM structure of Oat1 showing the extracellular and intracellular domains and the position of the substrate α-KG and the chloride ion. **c**, Top-down view of Oat1 highlighting the binding sites for α-KG (site 1) and chloride (site 2), respectively. **d**, Zoomed-in view of site 1, showing the main interactions formed with α-KG. Key residues interacting with the substrate are shown as sticks and hydrogen bonds are represented as dashed lines. Cryo-EM density for the ligand is shown (purple and threshold 0.415). **e**, Schematic of α-KG binding interactions. **f**, Cell-based transport assays for wild-type (WT) and mutant human OAT1. *n* = 15 independent experiments for the mutants and 80 for the wild type, errors shown are s.d. Inset shows IC_50_ for α-KG for wild-type and Gly227Ala mutant *n* = 4, data are mean ± s.d. Source data

OAT1 uses the outwardly directed α-KG gradient, maintained through the action of the sodium-dicarboxylate cotransporter (NaDC3) and via the Krebs cycle, to drive the uptake of organic anions from the blood across the basolateral membrane of the proximal tubules and the apical membrane of the choroid plexus^9–11^ (Fig. 1a). Many drugs are organic anions and thus concentrated in renal cells via SLC22 family members^12^, often resulting in adverse drug–drug interactions (DDI) and increased elimination^13–17^. Inhibition of OAT1 via probenecid is currently used to limit nephrotoxicity during antiretroviral treatment with cidofovir and tenofovir^18,19^. The polyspecific nature of OAT ligand recognition coupled with their role in renal clearance has made understanding how these transporters function an important part of drug development^20,21^. Increasing the range of specific inhibitors for OATs would enable more targeted intervention and clinical options for adjunct therapy to increase the concentration of drugs in the blood and reduce drug toxicity. However, the molecular basis by which OATs distinguish between ligands and inhibitors remains unclear, as does their transport mechanism, hampering efforts to design SLC22 subfamily-specific inhibitors.

## Results

### Cryo-EM structure of Oat1 bound to α-KG

**Extended Data Fig. 1:**
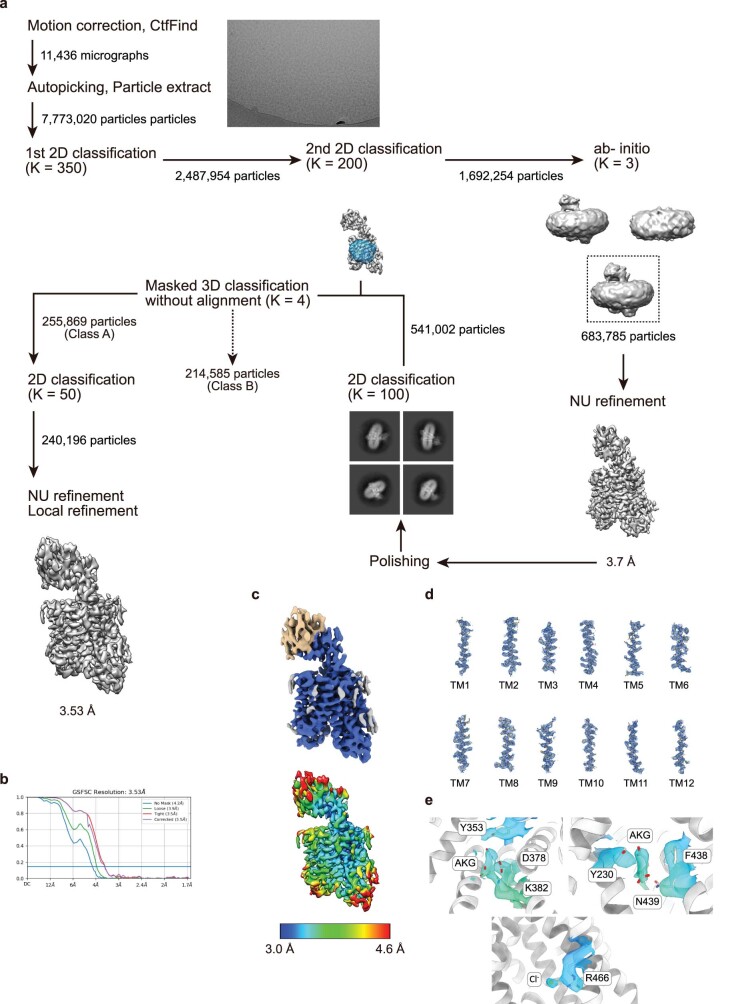
Cryo-EM processing workflow, showing local and global map quality for the Oat1–α-ketoglutarate sybody complex. **a**, Image processing workflow for Oat-sybody with α-KG. **b**, Gold-standard FSC curve used for global-resolution estimates within cryoSPARC. **c**, Local-resolution of reconstructed map as determined within cryoSPARC. Detergent density omitted for clarity. Sybody density is shown in wheat, Oat1 density blue and nonprotein density gray. **d**, Close-up view of map and side-chain density for transmembrane helices. **e**, Density for α-KG and side-chains coordinating the molecule. Density colour is the same resolution range given in **c**.

**Extended Data Fig. 2:**
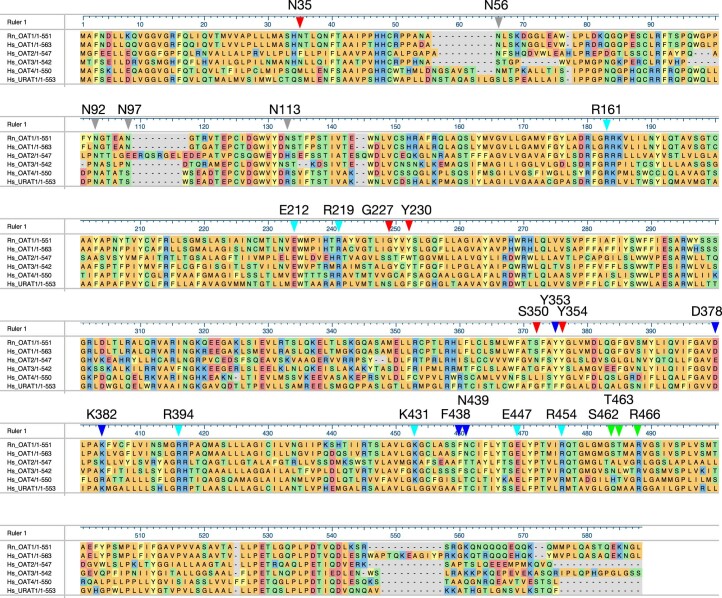
Sequence alignment of OAT1 homologues. The protein sequence of RnOat1 is shown aligned with human OAT1, 2, 3, 4 and URAT1. Both OAT1 and 3 utilise α-ketoglutarate to exchange a wide number of organic anions and for OAT3 also certain non-anionic compounds^58^. Whereas OAT2, in addition to transporting organic anions, can also transport various nucleobases including cGMP^59^. OAT4 has a more reduced organic anion substrate specificity which includes uric acid and estrone sulphate^60^. URAT1 is specific for uric acid uptake^61^. Highlighted are key important residues characterised within the study, blue – site 1, green – site 2, red – substrate selectivity, cyan salt bridge interacting residues, grey – N-glycosylation sites. Residue numbers are identical between human and rat sequences.

**Extended Data Fig. 3:**
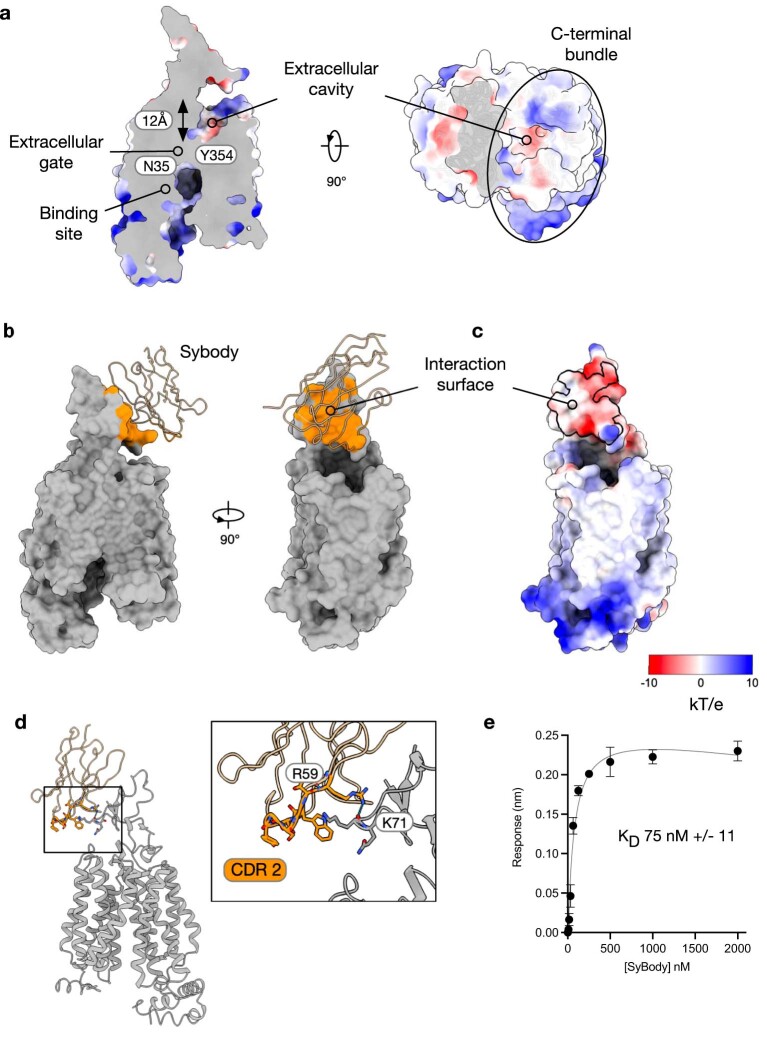
Analysis of Oat1-Sybody complex. **a**, Sliced through volume representation of Oat1 showing the electrostatic surface with key structural features highlighted. Electrostatic surface of Oat1. **b**, Surface display of Oat1 with cartoon display of the sybody binder. The buried solvent accessible surface area (>= 15 Å^2^) is highlighted in orange. **c**, Electrostatic surface representation of Oat1 with the buried surface area highlighted. **d**, The main polar interaction between the sybody and the extracellular domain of Oat1 occurs via the CDR2 loop, with Arg59 hydrogen bonding to the backbone carbonyl group of Lys71 in Oat1. **e**, The sybody used for structural determination has a K_D_ of 75 nM. n = 4 independent experiments, calculated mean ± s.d. is shown.

**Extended Data Fig. 4:**
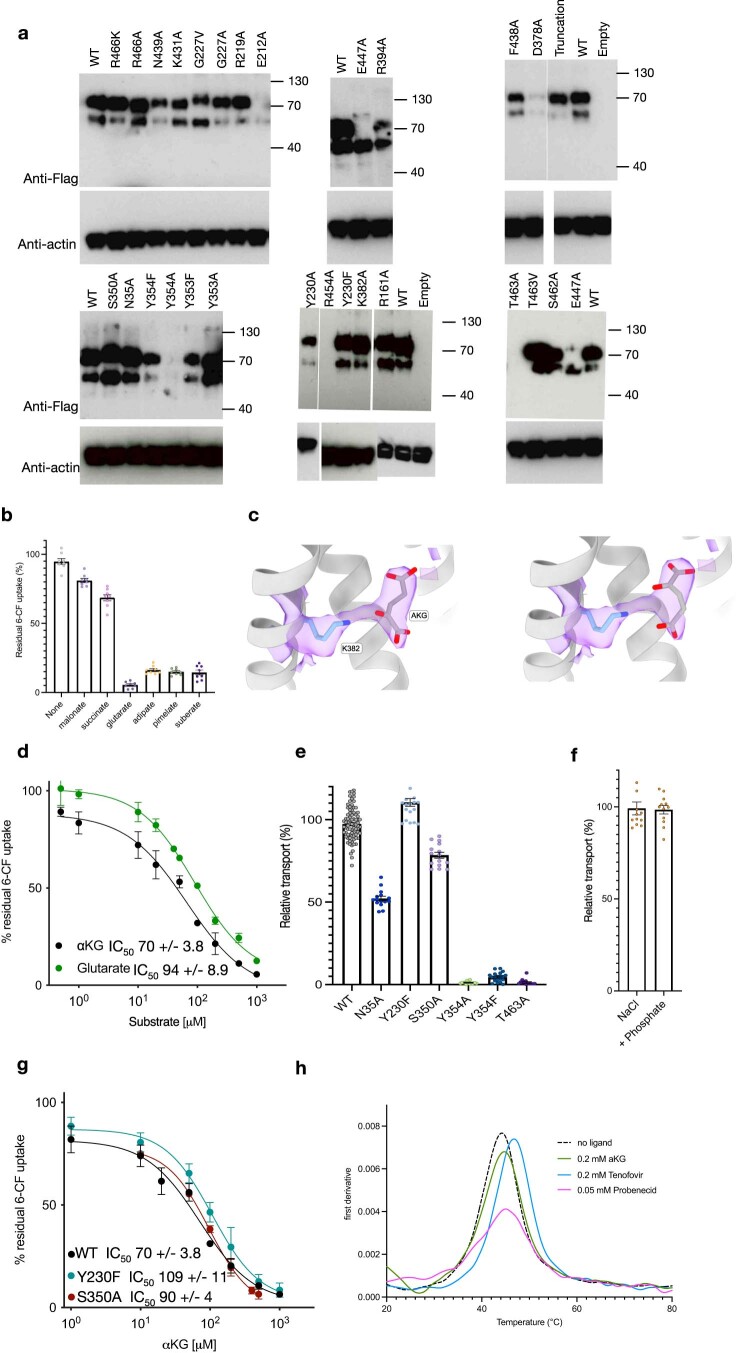
Functional characterisation of HsOAT1 and variants. **a**, Western blot of cell^−^ based assay using an anti-Flag antibody for HsOAT1 variants, complete with anti-β-actin control. HsOAT1 runs as two bands, which we interpret as an upper glycosylated band (indicating correct processing in the Golgi^62^) and a lower band. **b**, OAT1 shows a length specificity of dicarboxylate transport. Each dicarboxylate was added at 0.5 mM to the assay buffer and transport analysed after 8 minutes. n = 8 with error bars showing standard deviation. **c**, αKG modelled into the density observed in site 1. If αKG is flipped 180 °C (right), the fit to the density is worse. **d**, Representative IC_50_ curves of WT OAT1 for αKG or glutarate. Each concentration was repeated three times to calculate standard errors, and the whole experiment was repeated four times to calculate the mean IC_50_ and standard error. **e**, Analysis of transport activity of the variants of OAT1 used in the study but not included in the main figures. **f**, The presence of 10 mM sodium phosphate (+phosphate) has no effect on OAT1 transport. **g**, Representative IC_50_ curves for αKG for WT (also shown in Fig. 1f inset) and the Ser350Ala and Tyr230Phe variants. Each concentration was repeated three times to calculate standard errors, and the whole experiment was repeated three times to calculate the mean IC_50_ and standard error. **h**, nano-DSF^63^ analysis of Oat1 in the presence and absence of ligands. Source data

To understand the structural basis for α-KG recognition, we determined the cryogenic-electron microscopy (cryo-EM) structure of Oat1 from *Rattus norvegicus* in complex with a synthetic nanobody (sybody) at 3.53 Å (Fig. 1b, Extended Data Fig. 1a–e and Table 1). *Rn*Oat1, referred to as Oat1, shares 86% sequence identity (97% similarity) with the human homolog, referred to as OAT1 (Extended Data Fig. 2) and is used in drug development to analyze drug pharmacokinetics^22,23^. Oat1 adopts an inward open state with the canonical 12 transmembrane helices of the MFS fold, forming a binding site in the center of the membrane (Fig. 1c). A long polar cavity extends from the extracellular side of the membrane down toward the extracellular gate constructed by the packing of Asn35 (TM1) with Tyr354 (TM7), which seals a second large polar cavity that is open to the interior of the cell (Extended Data Fig. 3a). Unique features of the SLC22 family are the presence of an extracellular domain inserted between TM1 and TM2 and an intracellular domain between TM6 and TM7 (ref. ^24^). The extracellular domain contains four N-linked glycosylation sites, which are essential for the correct localization of Oat1 to the plasma membrane^25^ and is where the sybody binds to the transporter (Fig. 1b and Extended Data Fig. 3b–e). The intracellular domain consists of a four-helix bundle constructed of the N-terminal part of TM1, which packs against the C-terminal end of TM6 (Fig. 1b). The α-KG ligand was clearly identified from the cryo-EM maps and sits in a confined pocket, which we designate site 1, formed from TM5, TM7, TM8 and TM10 (Fig. 1c,d). Site 1 is conserved across the OAT family (Extended Data Fig. 2) and is located on one side of the binding site rather than in the center, where the most MFS ligands have been located to date^26–28^. As discussed below, the location of α-KG in site1 is due to a chloride ion binding site located roughly 11 Å away on the opposite side of the binding site, which we designate site 2 (Fig. 1c). α-KG sits close to Lys382 (TM8), which makes a strong hydrogen bond with the aldehyde group, and Tyr353 (TM7), which makes a hydrogen bond to the γ-carboxylate (Fig. 1e). Alanine mutants of both are nonfunctional indicating their importance for transport in human OAT1 (Fig. 1f and Extended Data Fig. 4). Located close (less than 6 Å) to α-KG are Tyr230 (TM5), Asp378 (TM8), Phe438 and Asn439 (TM10), all of which either negatively affect expression or transport as alanine mutants (Fig. 1f and Extended Data Fig. 4). Of note is the proximity of Gly227 (TM5), which sits within 3.3 Å of the α-carboxylate of the ligand, as this side chain is conserved within members of the OAT family that use α-KG to drive organic anion uptake (Extended Data Fig. 2). A Gly227Val mutant completely abolished transport in human OAT1, while the Gly277Ala mutant retains roughly 40% function. This suggests the glycine on TM5 is required in site 1 to create space for the ligand. Indeed, adding the extra methyl group in the alanine mutant negatively affects the IC_50_ (half-maximum inhibitory concentration) for α-KG (70 ± 3.8 μM for wild type versus 209 ± 23 μM for Gly227Ala) (Fig. 1f, inset). OAT1 demonstrates a length dependency for dicarboxylate uptake, with ligands containing fewer than five carbons not being recognized^29,30^ (Extended Data Fig. 4). α-KG is coordinated by Lys382 and Tyr353; the Tyr353Phe mutant retains roughly 50% activity, suggesting that direct interaction is not essential, while a bulky hydrophobic side chain is required at this position. The length dependency for a substrate is governed by the distance between Lys382 and Asp378, with shorter ligands unable to interact with these positions simultaneously and indicates a structural role for the ligand in facilitating transport.

**Table 1:** Cryo-EM data collection, refinement and validation statistics

	*Rn*Oat1 phosphate-bound state (EMD-16269), (PDB 8BVR)	*Rn*Oat1 α-KG-bound state (EMD-16280), (PDB 8BW7)	*Rn*Oat1 low-occupancy α-KG state (EMD-16977), (PDB 8OMU)	*Rn*Oat1 tenofovir-bound state (EMD-16270), (PDB 8BVS)
**Data collection and processing**				
Magnification	×105,000	×105,000	×105,000	×105,000
Voltage (kV)	300	300	300	300
Electron exposure (e^−^/Å^2^)	51	42	42	42
Defocus range (μm)	−1.0 to −2.0	−0.8 to −2.0	−0.8 to −2.0	−0.8 to −2.0
Pixel size (Å)	0.832	0.832	0.832	0.832
Symmetry imposed	*C*1	*C*1	*C*1	*C*1
Initial particle images (no.)	6,288,736	7,773,020	7,773,020	7,346,974
Final particle images (no.)	458,380	240,196	202,820	210,734
Map resolution (Å)	3.52	3.53	3.43	3.61
FSC threshold	0.143	0.143	0.143	0.143
Map resolution range (Å)	3.0–33	3.0–21	2.9–32	3.1–10
**Refinement**				
Initial model used	AlphaFold model (AF-O35956-F1)			
Model composition in the asymmetric unit				
Nonhydrogen atoms	4,806	4,748	4,737	4,799
Protein residues	617	612	612	617
Ligands^a^	3	2	0	2
Average *B* factors (Å^2^)				
Protein	66.7	81.5	57.6	106.6
Ligand	92.6	62.7		98.4
r.m.s.d.				
Bond lengths (Å)	0.004	0.003	0.003	0.003
Bond angles (°)	0.71	0.59	0.76	0.66
**Validation**				
MolProbity score		1.77	1.92	1.84
Clashscore		11.00	14.17	13.38
Poor rotamers (%)		0	0.98	0.78
Ramachandran plot				
Favored (%)	95.40	96.69	96.19	96.72
Allowed (%)	4.60	3.31	3.81	3.28
Outlier (%)	0	0	0	0

**Extended Data Fig. 5:**
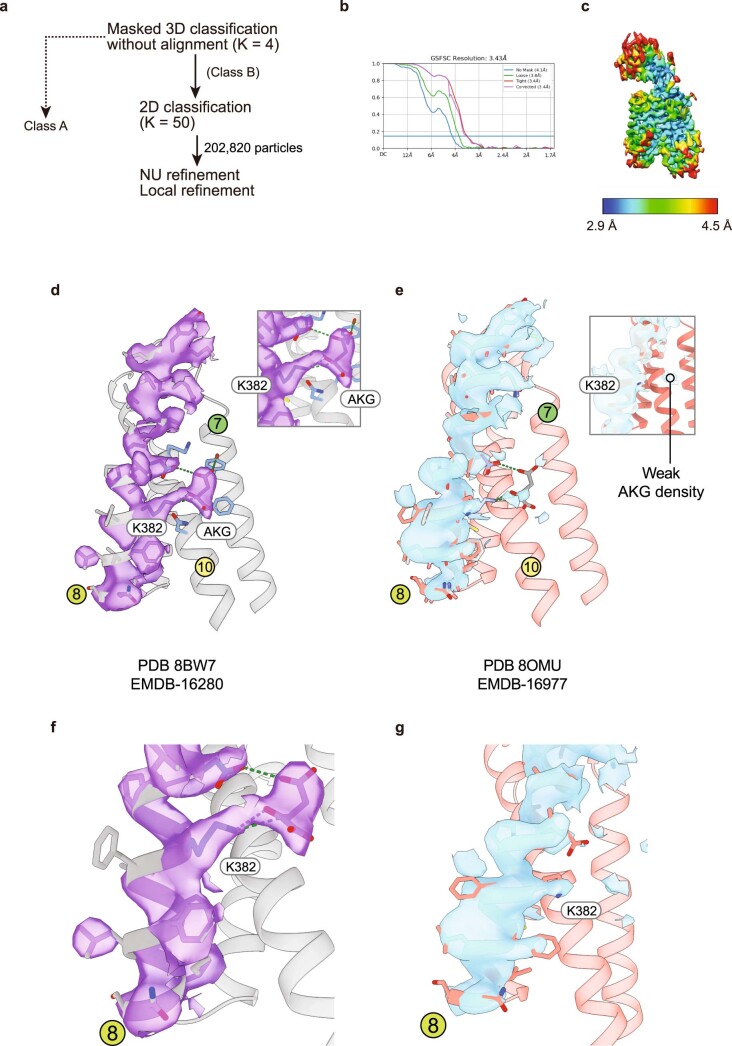
Cryo-EM processing workflow, showing local and global map quality for the low occupancy Oat1–α-ketoglutarate sybody complex. **a**, Image processing workflow for Oat-sybody with low occupancy α-KG. **b**, Gold-standard FSC curve used for global−resolution estimates within cryoSPARC. **c**, Local-resolution of reconstructed map as determined within cryoSPARC. Detergent density omitted for clarity. **d**, Density for TM8 and high occupancy α-KG from PDB 8BW7. Inset zoomed in view of density showing interaction Lys382 and the keto group of α-KG. **e**, Density for TM8 and low occupancy α-KG from PDB 8OMU. The α-KG molecule from d is shown for comparison. Inset zoomed in view of α-KG binding site (site 1) showing weak density for the ligand. **f**, and **g**, zoomed in views of TM8 from d and e respectively.

A key question in deciphering the mechanism of secondary active transporters is how ligand binding drives the conformational changes in the protein^28^. In the α-KG structure the backbone density for TM8 is less well resolved, particularly near Lys382, in comparison to neighboring helices (Extended Data Fig. 5). Additional classification of the α-KG dataset identified a second class of particles, resulting in a further structure of Oat1 at 3.43 Å resolution, where the α-KG and Cl^−^ densities are substantially reduced (Extended Data Fig. 5 and Table 1). The density around TM8 in the partially occupied Oat1 structure is stronger than observed in the α-KG bound state, and the Lys328 side chain adopts a different rotamer, pointing away from the ligand (Extended Data Fig. 5). The change in density around TM8 following α-KG binding indicates ligand induced structural changes, most likely through engagement with Lys382 and close positioning of the γ-carboxylate to Asp378. As discussed below, Asp378 forms a salt bridge network between TM8 and TM10, which involves Lys431 and must break to drive transport.

The ability of OAT1 to recognize glutarate, which lacks the keto group present in α-KG and is therefore symmetric, suggests that site 1 might accommodate α-KG in the opposite orientation, that is, flipped 180° relative to the one modeled (Fig. 1e). Modeled in this orientation the keto group would be too far away to interact with Lys382, instead replaced by the γ-carboxylate and, overall, there would be no discrimination between α-KG and glutarate (Extended Data Fig. 4c). However, the IC_50_ values for α-KG (70 ± 3.8 μM) and glutarate (94 ± 8.9 μM) reveals a slight preference for α-KG, indicating asymmetry in recognition of these two ligands (Extended Data Fig. 4d). Taken together, this result combined with the density around the carbonyl group in the ligand, supports a preferred orientation for α-KG in site 1, which is probably dominated by the interaction with Lys382.

### Allosteric regulation of OAT1 by chloride

**Fig. 2:**
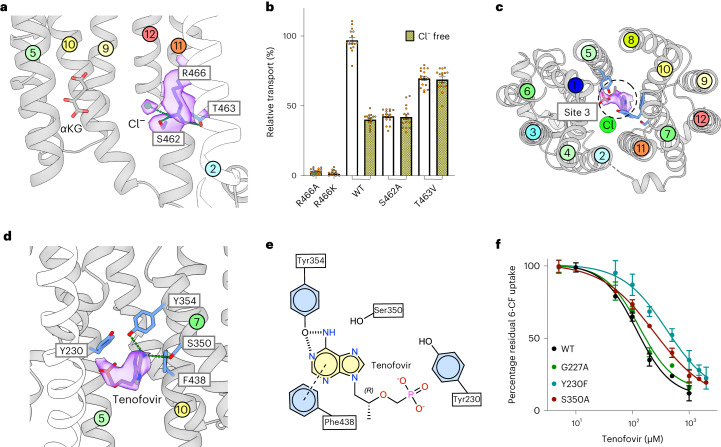
Molecular basis for chloride regulation and tenofovir recognition. **a**, Cryo-EM density for site 2 (purple and threshold 0.623). Interactions are indicated with green lines. **b**, Mutational analysis of site 2 and impact of chloride and chloride-free environments. *n* = 16, data are mean ± s.d. **c**, Top-down view of site 3 showing the Cryo-EM density observed for tenofovir (purple and threshold 0.329). **d**, Binding site showing residues interacting with tenofovir. Key residues interacting with the substrate are shown as sticks and hydrogen bonds are represented with dashed lines. Cryo-EM density for the ligand is shown (purple). **e**, Schematic of tenofovir binding interactions is shown in **d**. **f**, Effect of residue mutants on tenofovir recognition. IC_50_ values are the mean of three independent experiments; errors shown are s.d.

The kidneys play an important role in regulating plasma chloride levels, with the chloride concentration in the lumen of the proximal tubule increasing along the length of the nephron^31^. Chloride has been identified as an allosteric regulator of OAT1 and OAT3 but has no impact on OAT2 (refs. ^9,10,32^). Although a conserved arginine on TM11, Arg466, was implicated, the mechanism of chloride-based regulation remains unclear, given that all OAT transporters contain an equivalent arginine on TM11 (ref. ^30^) (Extended Data Fig. 2). In our structure, we observed a prominent density opposite α-KG in site 2 and sitting 3.5 Å from Arg466 (TM11), which we modeled as Cl^−^ (Fig. 2a). Neither an alanine mutant nor conservative substitution for lysine at this position in site 2 supported function (Fig. 2b). However, in the structure the presence of the Cl^−^ ion pushes Arg466 back into TM11, resulting in close interactions with Ser462 and Thr463. Confirming the importance of this interaction, neither the Ser462Ala nor Thr463Val mutants were chloride sensitive, whereas the wild type displays a marked dependence on this anion for maximal activity (Fig. 2b and Extended Data Fig. 4). Together, these results indicate the allosteric control of OAT1 transport via chloride results from the interaction network between the guanidinium group of Arg466 and the hydroxyls of Ser462 and Thr463. As discussed below, this network has important implications for gating dynamics in OAT1 and explains the allosteric regulation by chloride of organic anion transport in the kidney.

### Structural basis for tenofovir and probenecid recognition

**Extended Data Fig. 6:**
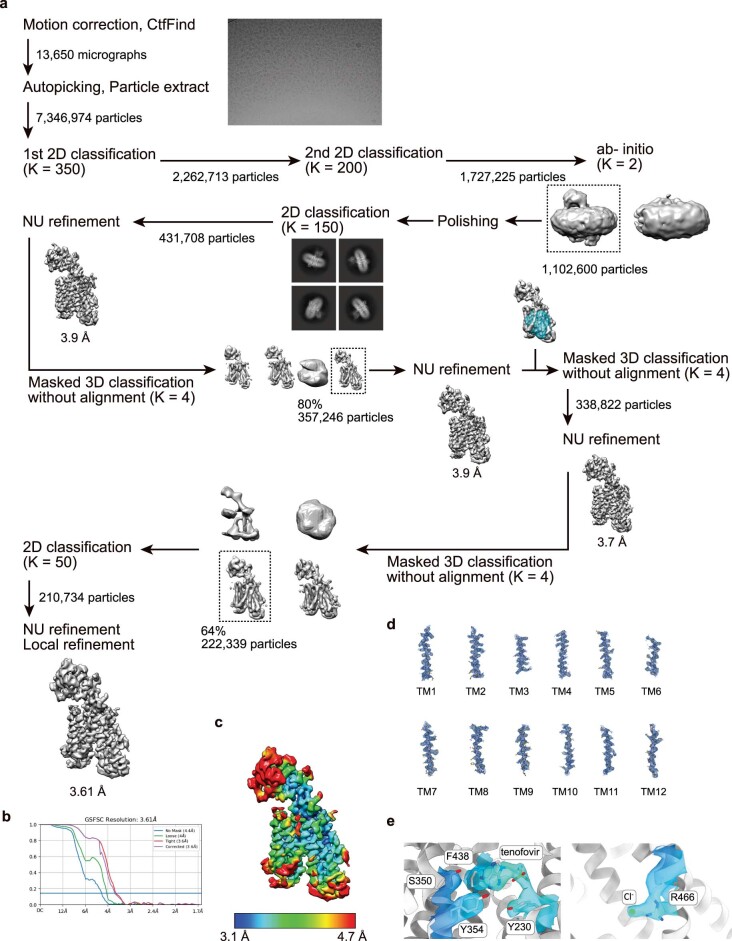
Cryo-EM processing workflow, showing local and global map quality for the Oat1-tenofovir sybody complex. **a**, Image processing workflow for Oat1-sybody with tenofovir. **b**, Gold-standard FSC curve used for global-resolution estimates within cryoSPARC. **c**, Local-resolution of reconstructed map as determined within cryoSPARC. Detergent density omitted for clarity. **d**, Close-up view pf map and side-chain density for transmembrane helices. **e**, Density for tenofovir and side-chains coordinating the molecule. Colour is the same range as **c**.

**Extended Data Fig. 7:**
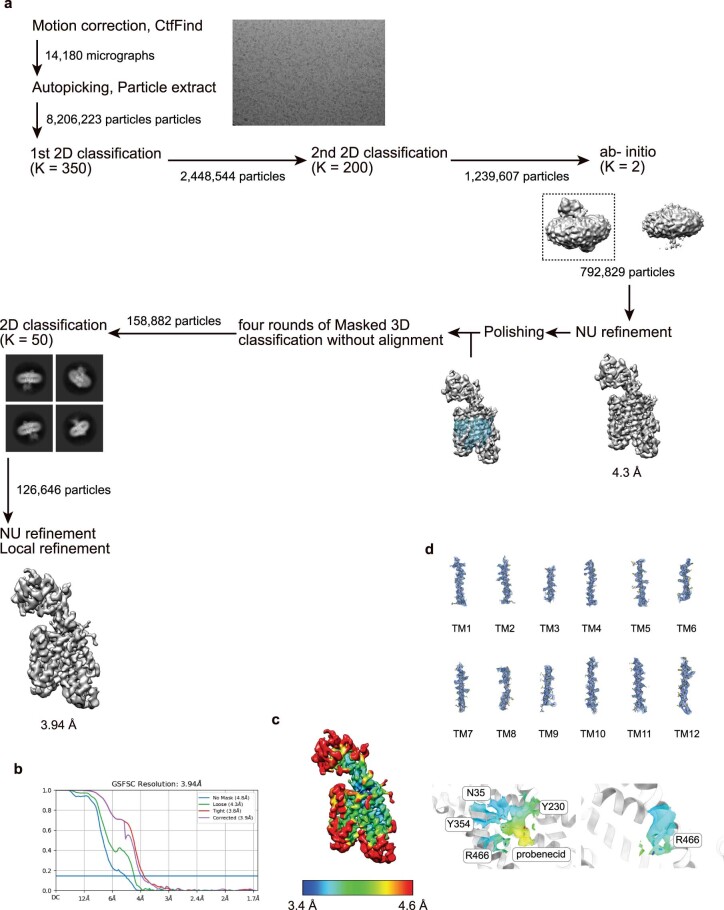
Cryo-EM processing workflow, showing local and global map quality for the Oat1-Probenecid sybody complex. **a**, Image processing workflow for RnOat1-sybody with probenecid. **b**, Gold-standard FSC curve used for global-resolution estimates within cryoSPARC. **c**, Local-resolution of reconstructed map as determined within cryoSPARC. Detergent density omitted for clarity. **d**, Close-up view pf map and side-chain density for transmembrane helices. **e**, Density for probenecid and side-chains coordinating the molecule. Colour is the same range as **c**.

To understand the molecular basis for drug transport and inhibition, we determined the structure of Oat1 in complex with the antiviral drug tenofovir and the archetypal OAT1 inhibitor probenecid at 3.61 and 4.01 Å, respectively (Tables 1 and 2 and Extended Data Figs. 6 and 7). Tenofovir is a member of a class of drugs called nucleotide reverse transcriptase inhibitors and is administered to treat hepatitis B and HIV^33^. Although tenofovir was clearly observed in the density map, the drug did not occupy site 1 (Fig. 2c). Instead, the drug was located centrally in a third pocket, which we designate site 3. The drug adopts a U-shape configuration with the nucleoside group making an aromatic π–π interaction with Phe438 (TM10). The amino group interacts with Tyr354 and sits close (roughly 3.5 Å) to Ser350 on TM7, while the phosphate group is positioned near Tyr230 (TM5), making an anion–π interaction (that is, the interaction of a negatively charged group with the positive electrostatic potential on the ring edge of an aromatic group^34^) (Fig. 2d,e). Aromatic interactions are a common mechanism for increasing substrate promiscuity in MFS binding sites^35^, with similar interactions observed in the related SLC22 family transporter OCT3 (refs. ^36,37^). Some of these side chains overlap with site 1, such as Phe438 and Tyr230, alanine substitutions of which are transport deficient in our assay (Fig. 1f). However, the Gly227Ala mutant had no discernible effect on tenofovir recognition (Fig. 2f), which along with the results for Ser350Ala on α-KG transport (discussed below) support the functional separation of sites 1 and 3 in the transporter. Within site 3, Tyr354Phe showed no activity, whereas Ser350Ala and Tyr230Phe retained sufficient function to analyze their contribution to tenofovir recognition (Fig. 2f and Extended Data Fig. 4). Tyr230Phe has previously been implicated in substrate specificity for OAT1 (ref. ^38^), and in our assays, the IC_50_ of this mutant for tenofovir was severely affected (120 ± 19 μM for wild type versus 450 ± 26 μM for Tyr230Phe). Unlike Tyr230, whose hydrophobic nature is conserved across the OAT family, Ser350 is not a conserved residue (Extended Data Fig. 2). However, an alanine mutant retained roughly 75% wild-type activity and its affinity for tenofovir doubled to 241 ± 11 μM (Fig. 2f) as opposed to having a negligible effect on α-KG uptake (Extended Data Fig. 4). These results confirm the functional separation of sites 1 and 3 and suggest that substrate promiscuity occurs due to the presence of distinct binding sites within the transporter.

**Table 2:** Cryo-EM data collection, refinement and validation statistics

	*Rn*Oat1 probenecid bound state (EMDB-16271) (PDB 8BVT)
**Data collection and processing**	
Magnification	×105,000
Voltage (kV)	300
Electron exposure (e^−^/Å^2^)	48
Defocus range (μm)	−1.0 to −2.0
Pixel size (Å)	0.832
Symmetry imposed	*C*1
Initial particle images (no.)	8,210,203
Final particle images (no.)	126,646
Map resolution (Å)	3.94
FSC threshold	0.143
Map resolution range (Å)	3.4–21
**Refinement**	
Model composition in the asymmetric unit	
Nonhydrogen atoms	4,517
Protein residues	623
Ligands^a^	1
Average *B* factors (Å^2^)	
Protein	136.9
Ligand	135.8
r.m.s.d.	
Bond lengths (Å)	0.009
Bond angles (°)	1.18
**Validation**	
MolProbity score	1.97
Clashscore	14.86
Poor rotamers (%)	0.23
Ramachandran plot	
Favored (%)	95.77
Allowed (%)	4.23
Outlier (%)	0

**Fig. 3:**
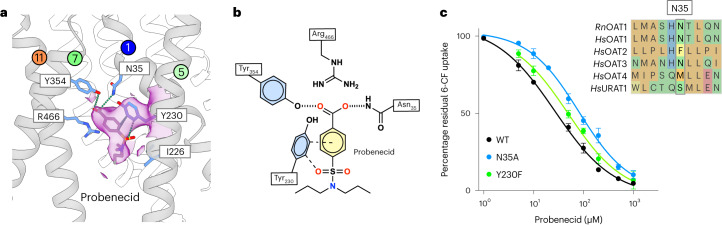
Recognition of probenecid. **a**, Cryo-EM density for probenecid in site 3 (purple and threshold 0.237). **b**, Schematic of probenecid interactions. **c**, Mutational analysis of probenecid binding site. IC_50_ values are the mean of three independent experiments; errors shown are s.d. Inset shows the sequence alignment of OATs with respect to the conservation of Asn35.

The OAT1 inhibitor probenecid was also observed in site 3. Although the global resolution was lower than for tenofovir and α-KG, the density for the aliphatic tails was distinct and used to orientate the drug in the binding site (Fig. 3a and Extended Data Fig. 7). Probenecid has a destabilizing effect on Oat1 (Extended Data Fig. 4h), likely contributing to the overall lower resolution. The sulfate group occupies the same position as the phosphate moiety in tenofovir. However, in the probenecid complex, Arg466 extends into site 3, displacing the chloride in site 2 (Fig. 3a,b). The carboxylate group also interacts via hydrogen bonds with extracellular gate residues Asn35 (TM1) and Tyr354 (TM7). The benzene ring interacts with Tyr230 in a 90° configuration, making a cation–π interaction. At the opposite end of the inhibitor, the aliphatic tails splay apart and sit near hydrophobic side chains Ile226 (TM5) and Phe442 (TM10). Given the lower resolution of the probenecid complex, we undertook a detailed analysis of the binding site to verify the observed interactions. An alanine mutant of Tyr354 failed to express, whereas the phenylalanine mutant was nonfunctional (Extended Data Fig. 4). However, the IC_50_ value for probenecid inhibition increased from 36 ± 8 μM for wild type to 59 ± 6 μM for Tyr230Phe and 89 ± 7 μM for Asn35Ala (Fig. 3c), highlighting their involvement in probenecid interactions. Probenecid is also an inhibitor of OAT3 and URAT1 (refs. ^29,39^), which contain an asparagine or serine, respectively, at the same position on TM1 (Fig. 3c, inset), but a poor inhibitor of OAT2 (ref. ^40^), which contains phenylalanine, indicating the importance of this site in regulating inhibitor interactions within the OAT family. Probenecid makes several additional interactions with the binding site compared with tenofovir and α-KG, directly coordinating TM1, TM7 and TM11 (Fig. 3b). These additional interactions, combined with the disruption of site 2 via the Arg466 salt bridge, would explain how this drug inhibits OAT transporters.

### Mechanism of OAT1 transport

**Fig. 4:**
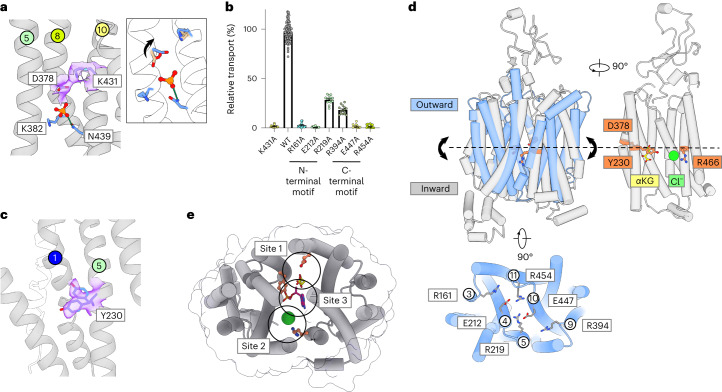
Mechanism of organic anion transport by Oat1. **a**, Cryo-EM density of the phosphate molecule observed in site 1 (purple and threshold 0.341). Inset shows that the movement of Asp378 was observed between the α-KG bound state (wheat) and phosphate-bound state (blue). **b**, Mutational analysis of salt bridge interaction between TM8 and TM10. *n* = 15 independent experiments for the mutants and 80 for the wild-type errors shown are s.d. **c**, Cryo-EM density for the two rotamer positions of Tyr230 (purple and threshold 0.341). **d**, Analysis of the inward-facing structure (gray) with an outward open model (blue) reveals how key residues, as well as α-KG and the Cl^−^ ion, align with where the helices pivot between states. Shown below are the salt bridge interactions identified from the outward open state that stabilize this conformation and form two ‘+−+’ motifs across the transporter. **e**, Oat1 contains three distinct ligand binding sites that could be targeted for selective inhibition.

**Extended Data Fig. 9:**
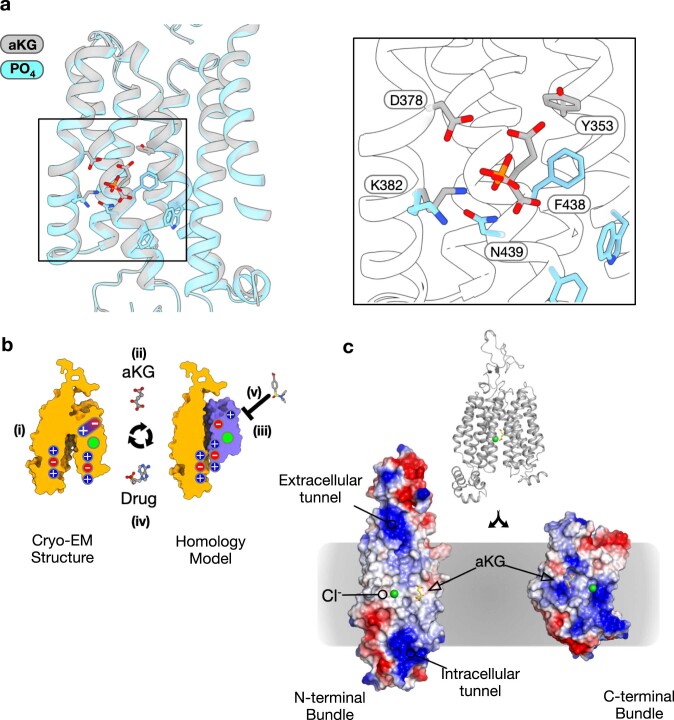
Superposition of phosphate molecule in Site 1 and the current model for alternating access transport in Oat1. **a**, Superposition of αKG and phosphate bound Oat1 (r.m.s.d. 0.633 Å over 496 C_α_ atoms). Inset left, zoomed in view of Site 1 with key residues labelled. **b, (i)** The inward open state of OAT1 is stabilised through the formation of the salt bridge between Asp378 (TM8) and Lys431 (TM10). **(ii)** α-KG binding to Site 1 results in the breakage of the Asp378-Lys431 salt bridge, enabling the transporter to switch to the outward open state. **(iii)** The outward open state is stabilised through the interactions between the ‘+−+’ motifs on TMs 4,5 and 10,11. **(iv)** Binding of organic anions to Site 1 or drugs to Site 3 stabilise the C-terminal domains and facilitate reorientation back to the inward open state, where the ligand is released into the cell. The chloride ion (green circle) in Site 2 facilitates transport by rigidifying the C-terminal bundle and may contribute to positioning α-KG in Site 1 and drugs into Site 3. (v) Probenecid inhibits OAT1 by interfering with the Asp378-Lys431 salt bridge lock, binding the extracellular gate residues (Asn35 & Tyr354) and may also disrupt Cl^−^ ion binding. **c**, Analysis of the electrostatic surface potential of the binding site in Oat1. The N-terminal bundle contains positively charged regions that lead into the main cavity from the extracellular and intracellular sides of the membrane respectively. These will facilitate anion entry and exit. However, the surfaces adjacent to Sites 1, 2 and 3 are notable for being hydrophobic and biochemically inert.

Alternating access transport within secondary active transporters occurs following the orchestrated movement of opposing gates that bracket a central ligand binding site within the protein^28^. Within the MFS, the extracellular gates are constructed from TM1 and TM2 from the N-terminal bundle, which pack against TM7 and TM8 from the C-terminal bundle^41,42^. In contrast, the opposing intracellular gate is formed by TM4 and TM5 packing against TM10 and TM11. Transported ligands, including ions, coordinate the gating helices across the N- and C-terminal bundles, often modulating salt bridge interactions to ensure that when one gate is open, the other is closed^43^. However, in Oat1, we observe three distinct ligand binding sites within the central cavity (sites 1–3), raising the question of how α-KG in site 1 drives the transport of organic anions into the cell. To address this question, we determined the structure of Oat1 in the absence of a ligand. The apo structure, determined at 3.52 Å (Table 1 and Extended Data Fig. 9a), contains density within site 1 close to Lys382 and Asn439 and occupying a similar position to the keto group of α-KG (Fig. 4a and Extended Data Fig. 9a). Given the buffer composition of the apo state contained high phosphate, unlike the ligand structures that had no phosphate, we modeled this density as a phosphate molecule. As phosphate has no impact on OAT1 transport we interpret this structure as the apo state (Extended Data Fig. 4f). Two further densities are observed, one interacting with the hydroxyl group of Tyr230 and the other sitting close to Phe438 and Phe442, which are likely to be additional phosphate molecules. We observed no obvious density in site 2. Comparing these two structures reveals no notable changes in the backbone positions (root mean squared deviation (r.m.s.d.) of 0.629 Å for 496 Cα atoms); however, two changes in the density around several conserved side chains were observed. Aspartate 378 (TM8) can now be modeled interacting with Lys431 (TM10) (Fig. 4a), whereas in the α-KG structure, Asp378 is rotated away, facing the α-KG ligand (Fig. 1d and Extended Data Fig. 1). Similar to Asp378, Lys431 is essential for function with an alanine mutant showing no activity (Fig. 4b). Therefore, the binding of α-KG appears to break the Asp378-Lys431 salt bridge interaction between two gating helices, TM8 and TM10. Phosphate, which OAT1 does not transport, is unable to break this interaction, underlining the importance not only of anion binding in site 1 but also the correct coordination of the ligand and confirming the length requirement discussed above (Extended Data Fig. 4b). These structural changes are consistent with and likely contribute to the increased disorder, and subsequent reduction in map quality, observed in TM8 following α-KG binding (Extended Data Fig. 5). The second observable difference is that Tyr230 (TM5) adopts two rotamer positions (Fig. 4c), whereas, in the ligand-bound structures, only one is observed (Extended Data Figs. 1e and 6e). Given the importance of a bulky hydrophobic side chain at this position (Fig. 1f) and the role of Tyr230 in discriminating between substrates (Fig. 2f), this residue likely plays a key structural role in stabilizing TM5 in response to ligand binding.

Tyr230, along with Asp378 (TM8) and Arg466 (TM11), all lie in the same plane within the binding site, which transects sites 1, 2 and 3. Positioned in the middle of the membrane and halfway down their respective gating helices, this axis provides the ideal position to allow the transporter to rock between outward and inward open states during transport. Using the structure of the outward open organic cation transporter, OCT3 (ref. ^36^), we generated a model for the outward-facing state of Oat1. This model supports the importance of the central axis as this locates the position of the pivot points between the outward- and inward-facing states (Fig. 4d). The outward-facing model also suggests two additional salt bridge interactions between Glu212 (TM4) and Arg454 (TM11) and Arg219 (TM5) and Glu447 (TM10) that are part of two symmetry-related ‘positive–negative–positive’ motifs on either side of the cytoplasmic entrance (Arg161–Glu212–Arg219 and Arg394–Glu447–Arg454) (Fig. 4d). Salt bridges are commonly used to stabilize the closed state of gating helices in MFS transporters^44^. Alanine mutants of this motif show reduced uptake for the first and last arginines in each motif to 20% versus wild type (Fig. 4b). By contrast, the side chains predicted to form salt bridge interactions (Glu212–Arg454 and Arg219–Glu447) either do not express showing a requirement for this residue in the stability of the protein or are essential for function.

## Discussion

Taken together, a model for OAT1 transport can be proposed (Extended Data Fig. 9b,c), where α-KG binding facilitates the structural transition from inward to outward open states through engagement with Lys382, breaking the Asp378-Lys431 salt bridge between TM8 and TM10 while simultaneously locking Tyr230 in one state that stabilizes TM5. The symmetry-related gating helix, TM11, is similarly stabilized through chloride binding to site 2, via Arg466, forming the intrahelical staple. The van der Waals radius (1.8 Å) of chloride likely facilitates the correct placement of ligands in sites 1 and 3 and explains its role in allosteric regulation. In contrast, the outward open state is stabilized through the salt bridge interactions in the opposing cytoplasmic positive–negative–positive motifs. The interconversion from outward to inward-facing states following drug binding on the extracellular side of the membrane is likely to be aided through the interactions with the large bulky side chains in site 3, which would pull the extracellular gate closed and push the intracellular gate open. Finally, the design of inhibitors for specific members of the SLC22 family would substantially expand clinical options for modulating drug pharmacokinetics^21,45^. The identification of functionally distinct sites within OAT1 (Fig. 4e) that not only discriminate between ligands but differ across SLC22 family members highlights new opportunities for family-specific inhibitor design.

## Methods

### Oligonucleotides

Oligonucleotides used in this study were as follows.

**Table:** 

HsOAT1_F	aacaacGCTAGCgccaccatggcctttaatgacctcc
HsOAT1_Rev_flag	gggcggCTCGAGTCACTTGTCGTCATCGTCTTTGTAGTCGCTGCCGCCgagtccattcttctcttgtgc
HsOAT1_trun_Rev_flag	gggcggCTCGAGTCACTTGTCGTCATCGTCTTTGTAGTCGCTGCCGCCtgggaccatatacttctggtgc
G227A_F	gcaccttgattgCctatgtctacagcctgg
G227A_R	ccaggctgtagacatagGcaatcaaggtgc
G227V_F	gcaccttgattgTctatgtctacagcctggg
G227V_R	cccaggctgtagacatagAcaatcaaggtgc
Y230A_F	gattggctatgtcgccagcctgggccagttcc
Y230A_R	ggaactggcccaggctggcgacatagccaatc
Y230F_F	gattggctatgtcTTcagcctgggccagttcc
Y230F_R	ggaactggcccaggctgAAgacatagccaatc
Y353A_F	ccactagctttgcaGCctatgggctggtcatgg
Y353A_R	ccatgaccagcccatagGCtgcaaagctagtgg
Y353F_F	ccactagctttgcaTTctatgggctggtcatgg
Y353F_R	ccatgaccagcccatagAAtgcaaagctagtgg
D378A_F	ctttggtgctgtggCcctgcctgccaagcttg
D378A_R	caagcttggcaggcaggGccacagcaccaaag
K382A_F	ggacctgcctgccGCgcttgtgggcttcc
K382A_R	ggaagcccacaagcGCggcaggcaggtcc
F438A_F	ctggctgcctccGCcaactgcatcttcc
F438A_R	ggaagatgcagttgGCggaggcagccag
N439A_F	ggctgcctccttcGCctgcatcttcctgtatactgg
N439A_R	ccagtatacaggaagatgcagGCgaaggaggcagcc
R466A_F	gcagcaccatggccGCagtgggcagcatcgtgagc
R466A_R	gctcacgatgctgcccactGCggccatggtgctgc
R466K_F	gcagcaccatggccAAagtgggcagcatcgtgagc
R466K_R	gctcacgatgctgcccactTTggccatggtgctgc
S462A_F	ggcatgggaatgggcGCcaccatggcccgagtg
S462A_R	cactcgggccatggtgGCgcccattcccatgcc
T463A_F	ggaatgggcagcGccatggcccgagtggg
T463A_R	cccactcgggccatggCgctgcccattcc
T463V_F	ggaatgggcagcGTcatggcccgagtggg
T463V_R	cccactcgggccatgACgctgcccattcc
S350A_F	gctgtggtttgccactGCctttgcatactatggg
S350A_R	cccatagtatgcaaagGCagtggcaaaccacagc
N35A_F	ggcttctcacgccaccctgcagaacttcac
N35A_R	gtgaagttctgcagggtggcgtgagaagcc
R161A_F	gcagacaggctaggcGCccggaaggtactcatc
R161A_R	gatgagtaccttccggGCgcctagcctgtctgc
K431A_F	gctgtgctggggGCgggttgtctggctgc
K431A_R	gcagccagacaacccGCccccagcacagc
E212A_F	gacactgaatgtggcgtggatgcccattca
E212A_R	tgaatgggcatccacgccacattcagtgtc
R219A_F	cccattcacacagcggcctgcgtgggcacc
R219A_R	ggtgcccacgcaggccgctgtgtgaatggg
R394A_F	caactccctgggtGCccggcctgcccag
R394A_R	ctgggcaggccggGCacccagggagttg
E447A_F	cctgtatactggggCactgtatcccacaatg
E447A_R	cattgtgggatacagtGccccagtatacagg
R454A_F	cccacaatgatcGCgcagacaggcatgg
R454A_R	ccatgcctgtctgcGCgatcattgtggg
Y354A_F	ctagctttgcatacGCtgggctggtcatggacc
Y354A_R	ggtccatgaccagcccaGCgtatgcaaagctag
Y354F_F	ctagctttgcatacTTtgggctggtcatggacc
Y354F_R	ggtccatgaccagcccaAAgtatgcaaagctag
RnOat1_F	atggccttcaatgacctcctgaaac
3′ GFP_Rev	taagcttgatatcgaattcctgcag

### Cloning, expression and purification of *Rn*Oat1

The gene encoding *R. norvegicus* Oat1 was inserted into the pFASTBAC vector upstream of a C-terminal tobacco etch virus (TEV) cleavable HIS_8_ tagged green fluorescent protein (GFP). The final construct used for structural determination lacked the last ten amino acids as C-terminal cleavage was observed during expression in insect cells of the full-length protein. Baculovirus was produced and used to infect 4 l of Sf9 cells (Gibco 11496015). Two days postinfection, cells were gathered, washed once in PBS and stored at −80 °C until required. Membranes were prepared from the cell pellet through lysis via sonication and subsequent ultracentrifugation. *Rn*Oat1 was purified from membranes to homogeneity using standard immobilized metal-affinity chromatography protocols in *n*-dodecyl-β-d-maltopyranoside (DDM) (Glycon D97002-C) detergent. Following TEV cleavage and a further nickel affinity step to remove the His-tagged TEV protease and GFP, the protein was subjected to size exclusion chromatography (Superdex 200 Increase, 28-9909-44; Cytiva) in a buffer consisting of either PBS or 20 mM Tris pH 7.5, 150 mM NaCl with 0.015% DDM. Biotinylated *Rn*Oat1 was produced by adding a C-terminal Avi-tag followed by a FLAG tag. The protein was purified using Flag affinity purification and size exclusion, following biotinylation by glutathione *S*-transferase-BirA overnight, the protein was subjected to a further size exclusion run.

### Cell-based 6-CF transport assays

To assay *Hs*OAT1 transporter activity the model substrate 6-carboxyfluorescein (6-CF)^46,47^ (Merck C0662) was used, which is recognized by OAT1 and taken into the cells in exchange for α-KG. The assay was validated to show that OAT1 exhibited the same substrate preferences as reported within the literature (Extended Date Fig. 10) and to analyze the effect of truncation of the C terminus.

HeLa cells (Merck 93021013-1VL) were maintained in Gibco DMEM (high glucose, GlutaMAX Supplement, pyruvate, 31966021) supplemented with 10% fetal bovine serum and 2 mM l-glutamine under 5% CO_2_ at 37 °C. The cell line was not authenticated and not tested for mycoplasma contamination. For transport assays, 1 × 10^5^ cells per well were seeded into 24-well plates and 24 h later transfected using FUGENE HD (Promega E2311) Transfection Reagent (0.4 μg DNA per 1 μl of FUGENE per well) with *Hs*OAT1 constructs containing a C-terminal FLAG tag in the vector pCDNA3.1 for 36 h. Expression of each mutant of *Hs*OAT1 was assessed through western blotting on membrane fractions using an anti-FLAG antibody 1:3,000 dilution (Merck F1804), using a loading control of anti-β-actin 1:5,000 dilution (Merck A2228) (Extended Data Fig. 4). For the assay, cells were washed once with buffer (typically 135 mM NaCl, 5 mM KCl, 1.2 mM MgCl_2_, 28 mM glucose and 25 mM HEPES, pH 7.2) before and additional wash in the same buffer for 1 min. 0.2 ml of assay buffer containing 10 μM 6-CF was added and removed after 8 min. The cells were washed three times with assay buffer before lysis by adding 0.2 ml 20 mM Tris pH 7.5, 0.2% Triton X-100 for 5 min. Then, 150 μl were removed to a 96-well plate, and the fluorescence was read (excitation 485, emission 528 nm) in a SpectraMax M3 plate reader. Background fluorescence was subtracted from cells transfected with the empty plasmid, and the data were normalized to 100%. To assess the activity of *Hs*OAT1 in the presence and absence of chloride, the buffer was 120 mM NaCl, 28 mM glucose and 25 mM HEPES, pH 7.2 or for chloride-free conditions, and 120 mM sodium gluconate was used. For these assays, the amount of 6-CF was increased to 20 μM and cells were collected after 8 min to assess the level of substrate taken up. To study the effect of ligands or inhibitors, the compound of interest was added to the assay buffer at the desired concentration. To calculate IC_50_ values for the compounds, the compound was added at different concentrations to the assay buffer containing 10 μM 6-CF and left on the cells for 8 min. Each concentration was repeated three times to calculate standard errors, and the whole experiment was repeated three times to calculate the mean IC_50_ and standard error.

### Thermal stability measurements

A Prometheus NT.48 (NanoTemper Technologies) was used to analyze thermal stability in the presence and absence of 0.1 mM ligands. A final concentration of 0.2 mg ml^−1^ protein in buffer (20 mM Tris pH 7.5, 150 mM NaCl and 0.015% DDM) was used and protein with ligand was incubated on ice for 20 min. Thermal measurements were carried out in a range from 20 to 90 °C with 1 °C min^−1^ steps. The resulting melting curves were generated by plotting the first derivative of the fluorescence ratio at 330/350 nm against temperature.

### Sybody selection

Sybody selection was performed against C-terminally Avi-Flag-tagged and biotinylated *Rn*Oat1 using methods described previously in ref. ^48^. A high-affinity sybody with a slow off rate, as measured using biolayer interferometry (Octet Red 384), was identified from the concave library. The sybody was cloned and expressed as a C-terminally MycHis tagged construct from the pSB-init vector (Addgene no. 110100), (pSB_Syb25 Addgene no. 197992).

### Cryo‐EM sample preparation and data acquisition

For the apo structure, *Rn*Oat1 purified in PBS was mixed with a 1.2 molar excess of the sybody and incubated on ice for at least 60 min before grid preparation. For drug-bound complexes *Rn*Oat1 in Tris/NaCl was mixed with the compound for 1 h, and the sybody was added after this for a further hour, α-KG was added at a final concentration of 1.2 mM, tenofovir at 0.6 mM and probenecid at 0.25 mM. The complex (5 mg ml^−1^) was adsorbed to glow-discharged holey carbon-coated grids (Quantifoil 300 mesh, Au R1.2/1.3) for 10 s. Grids were then blotted for 3 to 6 s at 100% humidity (8 °C) and frozen in liquid ethane using a Vitrobot Mark IV (Thermo Fisher Scientific). Data were collected in counted super-resolution mode on a Titan Krios G3 (FEI) operating at 300 kV with a BioQuantum imaging filter (Gatan), and K3 direct detection camera (Gatan) at ×105,000 magnification, physical pixel size of 0.832 Å. Then, 12,071 videos (5,997 and 6,074 videos in datasets 1 and 2, respectively) were collected for Oat1-Syb in the phosphate-bound state at a dose rate of 17 e^−^/Å^2^ per s, exposure time of 3.00 s, corresponding to a total dose of 51 e^−^/Å^2^ over 40 fractions. For the Oat1 α-KG bound state, 11,436 videos were collected at a dose rate of 14 e^−^/Å^2^ per s, an exposure time of 3.00 s, corresponding to a total dose of 42 e^−^/Å^2^ over 40 fractions. For the Oat1-tenofovir bound state, 13,650 videos were collected at a dose rate of 14 e^−^/Å^2^ per s, an exposure time of 3.00 s, corresponding to a total dose of 42 e^−^/Å^2^ over 40 fractions. For the Oat1-probenecid bound state, 13,650 videos were collected at a dose rate of 16 e^−^/Å^2^ per s, an exposure time of 3.0 s, corresponding to a total dose of 48 e^−^/Å^2^ over 40 fractions.

### Cryo-EM data processing and model building

Initial micrograph processing was performed in real-time using the SIMPLE pipeline^49^ using SIMPLE-unblur for patched (15 × 10) motion correction, SIMPLE-CTFFIND for patched contrast transfer function estimation and SIMPLE-picker for particle picking and particle extraction. All subsequent processing was performed in either cryoSPARC^50^ or RELION-3.1 (ref. ^51^) using the csparc2star.py script within UCSF pyem^52^ to convert between formats. Resolution estimates were derived from gold-standard Fourier shell correlations (FSCs) using the 0.143 criteria calculated within cryoSPARC. Local-resolution estimations were calculated within cryoSPARC.

For *Rn*Oat1 α-KG (Extended Data Fig. 1), the extracted 7,773,020 particles were subjected to two rounds of 2D classification and selected 2,487,954 particles were classified by three classes ab initio reconstruction. Using the apo state as the reference map (described below), 683,785 particles belonging to the *Rn*Oat1–Syb map were used for nonuniform refinement (8 Å initial low-pass) to yield a 3.7 Å map. These particles were Bayesian polished and classified in 2D to generate a subset of 541,002 cleaned and polished particles. To obtain *Rn*Oat1–Syb particles with α-KG, these particles were subjected to four classes of alignment-free classification with a mask around the binding site in RELION. One class of the classification was selected, and subsequently the remaining junk was removed by 2D classification. The remaining particles (240,196) were used for nonuniform and local refinements in cryoSPARC, and the final *Rn*Oat1–Syb with α-KG map was determined at 3.53 Å, based on the FSC = 0.143 criteria. Another class of alignment-free classification were selected, and similar steps were performed. The remaining particles (202,820) were used for nonuniform and local refinements in cryoSPARC, and the final *Rn*Oat1–Syb with low-occupancy α-KG map was determined at 3.43 Å, based on the FSC = 0.143 criteria.

**Extended Data Fig. 10:**
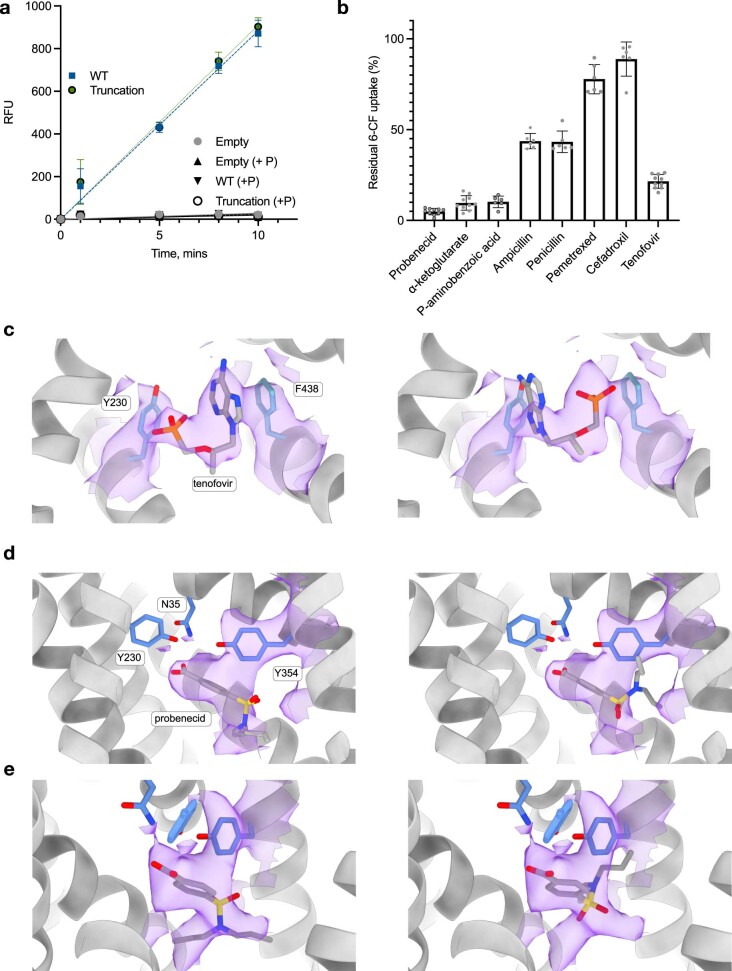
Functional characterisation of HsOAT1 using 6-carboxyfluorescein. **a**, The fluorescent model substrate 6-carboxyfluorescein was used to assess the function of HsOAT1 overexpressed in HeLa cells. The substrate was taken up by the cells over expressing WT HsOAT1 (WT) to a high level compared to cells transfected with the empty vector (Empty). The amount of compound also increased over time and was fully inhibited through the addition of 0.1 mM pemetrexed (+P) to the assay buffer. A truncated version which lacked the last 10 amino acids (Truncation), akin to the construct used for structural determination, did not show any difference in uptake behaviour to WT protein. The C-terminal region of OAT1 had also been previous reported to be non-required for transport^64^. n = 4 independent experiments with error bars showing standard deviation. **b**, The 6-CF assay shows a similar substrate specificity for OAT1 as reported within the literature^20^ extending from known good substrate (α-ketoglutarate, p-aminobenzoic acid), to poor substrates (pemetrexed, cefadroxil). All substrates were added at 0.5 mM to the buffer and the data shown is uptake after 8 minutes. n = 6 independent experiments with error bars showing standard deviation. **c**, Tenofovir modelled into the density observed in site 3 as described in the study (Left). If the ligand is flipped 180 °C (right), the fit to the density and favourable interactions are worse. **d** and **e**, Probenecid modelled into the density observed in site 3 as described in the study (Left). If the ligand is flipped 180 °C (right), the fit to the density and favourable interactions are worse.

For *Rn*Oat1–Syb with tenofovir (Extended Data Fig. 5), the extracted 7,346,974 particles were subjected to three rounds of alignment-free classification (whole, pocket and whole masks in first, second and third rounds, respectively) in RELION, the remaining junk particles were removed by 2D classification. Then 1,102,600 particles belonging to *Rn*Oat1–Syb were Bayesian polished and classified in 2D to generate a subset of 431,708 cleaned and polished particles, which yielded a 3.9 Å map. Particles (210,734) belonging to *Rn*Oat1–Syb with tenofovir were used for nonuniform and local refinements in cryoSPARC, and the final map was determined at 3.61 Å, based on the FSC = 0.143 criteria. The adenine moiety of tenofovir fits the nonprotein density, with an aromatic π–π interaction by Phe438, and the further additional density corresponding to the phosphonomethoxypropy moiety was observed between the adenine moiety and Tyr230 (Extended Data Fig. 10). For *Rn*Oat1–Syb with probenecid (Extended Data Fig. 6), the extracted 8,206,223 particles were subjected to two rounds of 2D classification, and selected particles were classified by two classes ab initio reconstruction. Then 792,829 particles belonging to *Rn*Oat1–Syb were Bayesian polished in RELION, then were subsequently subjected to four rounds of alignment-free classification, using a mask around the binding site in RELION, and remaining junks were removed by 2D classification. Particles (107,118) belonging to *Rn*Oat1–Syb with probenecid were used for nonuniform and local refinements in cryoSPARC, and the final map was determined at 4.01 Å, based on the FSC = 0.143 criteria. Model and restrain files were generated by PHENIX elbow package^53,54^. The benzoic moiety of tenofovir fit the nonprotein density observed near Tyr230, and the further additional density corresponding to the dipropylsulfamoy moiety was observed near the benzoic moiety (Extended Data Fig. 10).

For the *Rn*Oat1 phosphate state (Extended Data Fig. 7), in dataset 1, all extracted particles were classified by two rounds of 2D in cryoSPARC and four classes ab initio reconstruction in cryoSPARC was performed by selected 969,323 particles derived from the second round of 2D classification. Subsequently, particles belonging to a putative *Rn*Oat1–Syb map were reconstituted by the second round of ab initio. Next, all extracted 6,288,736 particles from both datasets 1 and 2 were subjected to one round of 2D classification in cryoSPARC and selected 3,021,945 particles were used for heterogeneous refinement (10 Å initial low-pass) under three junk (first round ab initio) and one *Rn*Oat1–Syb (second round of ab initio) maps. Particles in the *Rn*Oat1–Syb class were subjected to one round of 2D classification in cryoSPARC. Subsequently, 585,341 particles were reconstituted into a 3D map by nonuniform refinement (8 Å initial low-pass), using the second-round ab initio map as the reference, to yield a 4.3 Å map. These particles were Bayesian polished^51^ and classified in 2D to generate a subset of 543,720 cleaned and polished particles, which yielded a 3.7 Å map in nonuniform refinement. These particles were subjected to alignment-free classification with a whole protein mask in RELION, and selected 92% (502,704) particles were subjected to 2D classification in cryoSPARC to remove remaining junk particles. Then 458,380 particles belonging to *Rn*Oat1–Syb were used for nonuniform (8 Å initial low-pass) and local (8 Å initial low-pass) refinements, and the final map was determined at 3.52 Å based on the FSC = 0.143 criteria.

The AlphaFold^55^ model of *Rn*Oat1 (ID AF-O35956-F1) was manually fitted in the phosphate-bound state map on Chimera to generate the initial model. After model fitting, the models were manually readjusted using COOT^56^ and refined using PHENIX^53^. The model and restraint information for tenofovir and probenecid were generated by the eLBOW program^54^. The figures depicting the molecular structures were prepared using Chimera^57^, PyMOL (The Pymol Graphics System, v.2.0; Schrödinger) and CueMol.

### Reporting summary

Further information on research design is available in the Nature Portfolio Reporting Summary linked to this article.

## Online content

Any methods, additional references, Nature Portfolio reporting summaries, source data, extended data, supplementary information, acknowledgements, peer review information; details of author contributions and competing interests; and statements of data and code availability are available at 10.1038/s41594-023-01039-y.

### Supplementary information

Reporting Summary.

### Source data

Source Data Fig. 1 Statistical source data, each figure as a separate tab.

Source Data Extended Data Fig. 4 Uncropped western blots.

## Figures and Tables

**Extended Data Fig. 8:**
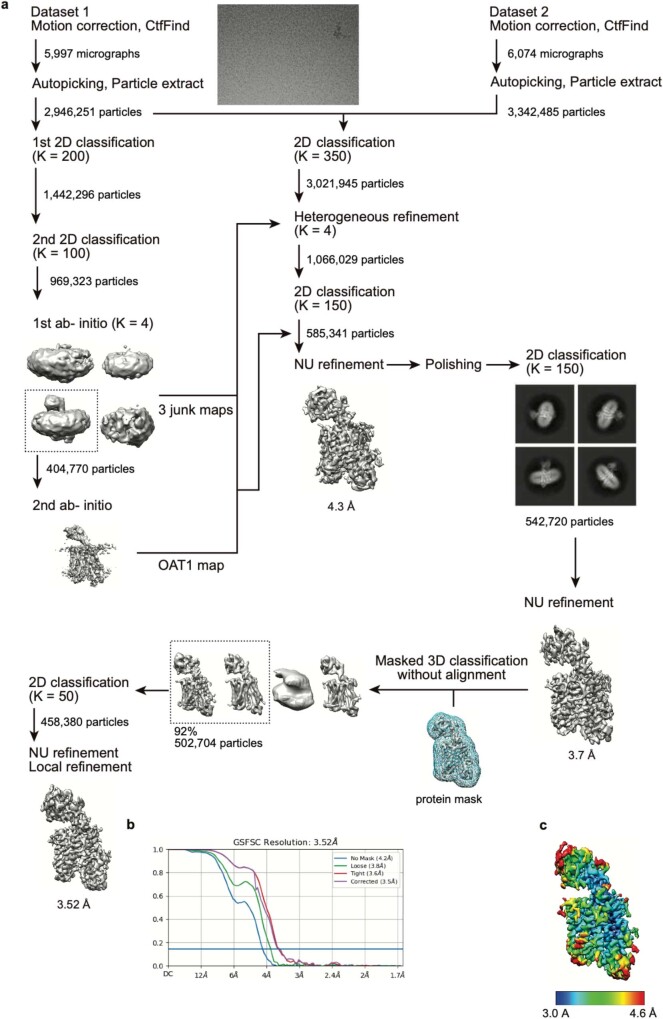
Cryo-EM processing workflow, showing local and global map quality for the apo-Oat1 sybody complex. **a**, Image processing workflow for OAT1-sybody in the absence of ligand. **b**, Gold-standard FSC curve used for global-resolution estimates within cryoSPARC. **c**, Local-resolution of reconstructed map as determined within cryoSPARC. Detergent density omitted for clarity.

## Data Availability

Coordinates for the structures have been deposited in the Protein Data Bank (PDB) under accession codes PDB 8BW7 (Oat1-Syb-α-KG), 8OMU (Oat1-Syb-low-occupancy α-KG), 8BVS (Oat1-Syb-tenofovir), 8BVT (Oat1-Syb-Probenecid) and 8BVR (Oat1-Syb-Phosphate). The electron microscopy volumes have been deposited in the Electron Microscopy Data Bank (EMDB) under accession codes EMD-16280 (Oat1-Syb-AKG), EMDB-16977 (Oat1-Syb-low-occupancy α-KG), EMD-16270 (Oat1-Syb-tenofovir), EMD-16271 (Oat1-Syb-probenecid) and EMD-16269 (Oat1-Syb). Source data for all uptake assays are provided with this paper. For all other source data, please contact S.N. All reasonable requests for source data will be actioned with an appropriate MTA.
