# Supplementary figures and images for: Molecular basis for selective uptake and elimination of organic anions in the kidney by OAT1
Source: Nat Struct Mol Biol. 2023 Jul 23;30(11):1786–93. doi: 10.1038/s41594-023-01039-y (PMC10643130; doi:10.1038/s41594-023-01039-y)

## Source data for Extended data figure 4 - Uncropped Westerns

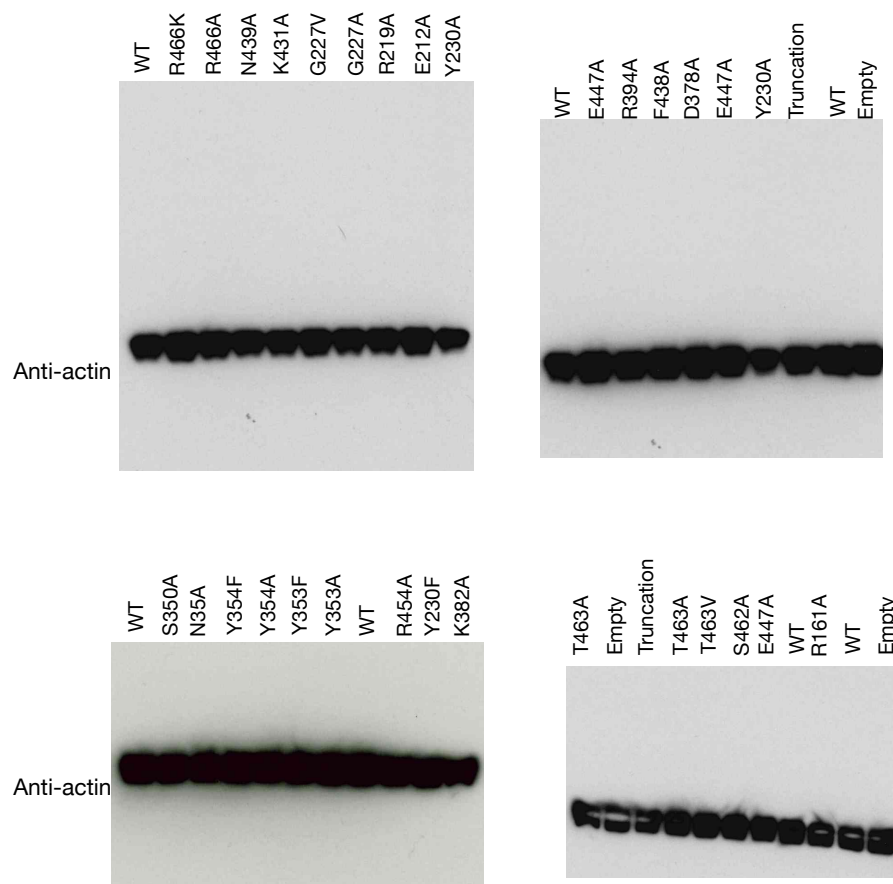

Supplement: Supplementary file 3 — Uncropped western blots. [file 41594_2023_1039_MOESM3_ESM.pdf]
